# Spatio-temporal Transmission and Environmental Determinants of Schistosomiasis Japonica in Anhui Province, China

**DOI:** 10.1371/journal.pntd.0003470

**Published:** 2015-02-06

**Authors:** Yi Hu, Rui Li, Robert Bergquist, Henry Lynn, Fenghua Gao, Qizhi Wang, Shiqing Zhang, Liqian Sun, Zhijie Zhang, Qingwu Jiang

**Affiliations:** 1 Department of Epidemiology and Biostatistics, School of Public Health, Fudan University, Shanghai, China; 2 Key Laboratory of Public Health Safety, Ministry of Education, Shanghai, China; 3 Laboratory for Spatial Analysis and Modeling, School of Public Health, Fudan University, Shanghai, China; 4 Ingerod, Brastad, Sweden; 5 Biomedical Statistical Center, Fudan University, Shanghai, China; 6 Anhui Institute of Parasitic Diseases, Wuhu, China; National Institute of Parasitic Diseases China CDC, CHINA

## Abstract

**Background:**

Schistosomiasis japonica still remains of public health and economic significance in China, especially in the lake and marshland areas along the Yangtze River Basin, where the control of transmission has proven difficult. In the study, we investigated spatio-temporal variations of *S. japonicum* infection risk in Anhui Province and assessed the associations of the disease with key environmental factors with the aim of understanding the mechanism of the disease and seeking clues to effective and sustainable schistosomiasis control.

**Methodology/Principal Findings:**

Infection data of schistosomiasis from annual conventional surveys were obtained at the village level in Anhui Province, China, from 2000 to 2010 and used in combination with environmental data. The spatio-temporal kriging model was used to assess how these environmental factors affected the spatio-temporal pattern of schistosomiasis risk. Our results suggested that seasonal variation of the normalized difference vegetation index (NDVI), seasonal variation of land surface temperature at daytime (LSTD), and distance to the Yangtze River were negatively significantly associated with risk of schistosomiasis. Predictive maps showed that schistosomiasis prevalence remained at a low level and schistosomiasis risk mainly evolved along the Yangtze River. Schistosomiasis risk also followed a focal spatial pattern, fluctuating temporally with a peak (the largest spatial extent) in 2005 and then contracting gradually but with a scattered distribution until 2010.

**Conclusion:**

The fitted spatio-temporal kriging model can capture variations of schistosomiasis risk over space and time. Combined with techniques of geographic information system (GIS) and remote sensing (RS), this approach facilitates and enriches risk modeling of schistosomiasis, which in turn helps to identify prior areas for effective and sustainable control of schistosomiasis in Anhui Province and perhaps elsewhere in China.

## Author Summary

Schistosomiasis japonica is one of the most serious parasitic diseases in China. It is estimated that more than 50 million people are still at risk, especially those living in the lake and marshland areas along the Yangtze River Basin. The Chinese government has made great efforts to implement schistosomiasis control programs since 1950s. The latest, major two programs are the 10-year World Bank Loan Project (WBLP) terminated in 2001, which was based on large-scale chemotherapy, and the national integrated control strategy implemented since 2005, which was aimed at reducing the roles of bovines and humans as infection sources. Based on spatio-temporal analyses of the *S*. *japonicum* infection prevalence data during 2000–2010 in Anhui Province, we found schistosomiasis prevalence remained at a low level but the spatial distribution of the disease became widely scattered at the later stage of the study period, suggesting that the integrated program could not fully effectively reduce the spatial extent of schistosomiasis risk. To achieve an effective and sustainable control strategy, we emphasize the need to control snail habitats within areas of high schistosomiasis risk.

## Introduction

Schistosomiasis, caused by trematode worms belonging to the *Schistosoma* genus [1], remains a serious public health problem worldwide [2]. More than 200 million people in approximately 76 countries are affected by the disease with at least a loss of 1.7 to 4.5 million disability-adjusted life years (DALYs) [3] and probably considerably more [4]. The true global burden of schistosomiasis japonica alone has been shown to be between four to 30 times greater than previously expected [5]. Of the three main schistosome species, *Schistosoma japonicum* is responsible for human and animal infections in southern China, large parts of The Philippines, and limited foci in Indonesia [6].

According to geographical patterns of the endemic areas and ecological characteristics of the vector snail, schistosomiasis endemic regions in China have been classified into three types: lake and marshland regions, plain regions with water-way networks, and hilly and mountainous regions [7]. Compared to the other two regions, control of the disease in the lake and marshland regions has proved to be difficult due to vast areas of *Oncomelania hupensis* habitats [8], and over 80% of schistosomiasis cases occurred in these regions [9]. With the Yangtze River passing across the province and the presence of large amounts of wet land, Anhui presents suitable environmental conditions for the formation of lake-and-marsh endemic regions.

The transmission of schistosomiasis is closely associated with the distribution of the intermediate host snail, which largely depends on environmental conditions such as vegetation coverage, temperature of freshwater, and quality and humidity of the soil [10,11]. In the lower reaches of the Yangtze River Basin, snails are distributed along the shore of rivers or lakes. The regular tide, together with large amount of marshland, provides an ideal environment for snail growth and reproduction, which can be characterized by “land in winter, water in summer” [12]. Under such favorable physical conditions, there is a concern that snails might explode and spread again, possibly giving rise to extensive re-emergence of infections among humans and domestic animals in the basin.

Techniques of geographic information system (GIS) and remote sensing (RS), combined with geostatistics, have been widely used in modeling the burden of schistosomiasis over the past decades [12,13,14,15,16]. However, most of the previous work only considered spatial correlations of prevalence data, and only a few attempts have been made to investigate spatio-temporal correlations and to assess how environmental factors affect these correlations. In this study, we aim to investigate changes in the spatio-temporal pattern of schistosomiasis in Anhui Province of China to better understand how environmental factors affect the changes of the disease, using a spatio-temporal kriging model [17]. This method offers a variety of techniques to make optimal use of measurement information for interpolating attributes in space and time, and has been applied over the last decade in such diverse scientific disciplines as environmental science [18], meteorology [19], and soil science [20].

## Materials and Methods

In this study, annual parasitological data obtained through standardized surveys during 2000–2010 are firstly analyzed with several environmental factors, and then the changes in spatio-temporal pattern of schistosomiasis are investigated.

### Approach and study area

**Figure 1:**
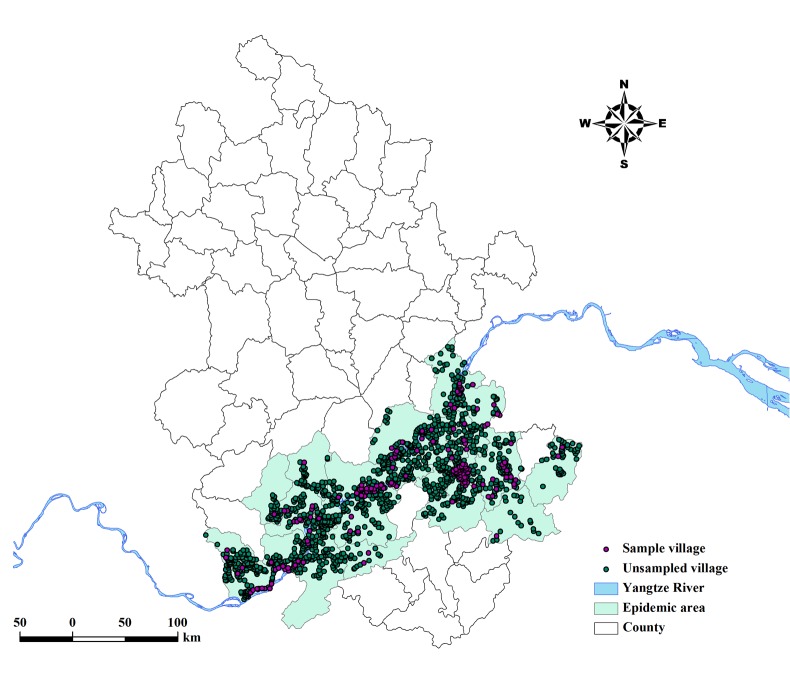
Location of sample villages in the study area. The geographical layer of water body was overlaid. The map was created using the AcrGIS software (version 10.0, ESRI Inc., Redlands, CA, USA).

In the present study, we integrate data sourced by application of GIS and RS with spatio-temporal kriging modeling to assess the schistosomiasis risk. The analysis is conducted at the village level with the study area located in Anhui in eastern China (Fig. 1). Anhui is a province spanning approximately 139,600 square kilometers and with a population of 59.9 million (2012). Most of the province is very flat, with a series of hills and ranges covering southwestern and southeastern Anhui. Major rivers include the Huaihe River in the north and the Yangtze River in the south. The province enjoys a subtropical humid monsoon climate. Plum rains occur in June and July and may cause flooding.

### Parasitological data

The *S*. *japonicum* infection prevalence data during 2000–2010 were obtained from cross-sectional surveys carried out by health professionals of the Anhui Institute of Parasitic Diseases (AIPD). The data were collected annually through village-based field surveys using a two-pronged diagnostic approach: screening by a serological test of all residents 5 to 65 years old and confirmed by Kato-Katz stool examination [21]. The results were reported to AIPD via county offices. The database used in this study consisted of 161 sample villages located in 24 schistosome-endemic counties, which were selected from the database of annual schistosomiasis surveys with the criteria that the village should be surveyed every year and the examined people should be over 100. Fig. 1 shows the locations of the sample villages in the endemic area.

### Ethics statement

Approval for oral consent and other aspects of this survey was granted by the Ethics Committee of Fudan University (ID: IRB#2011–03–0295). Written informed consent was also obtained from all participants.

### Environmental data

**Climatic data**. The climatic data include normalized difference vegetation index (NDVI) and land surface temperature at daytime (LSTD) and night (LSTN). All 8-day global 1 km products for LST and monthly global 1 km products for NDVI during the period 2000–2010 that covered Anhui Province were downloaded from the Level 1 and Atmosphere Archive and Distribution System (http://ladsweb.nascom.nasa.gov/data/search.html). These images were georeferenced and subsetted in ENVI software (version 5.0, Research System Inc.; Boulder, CO, USA). AcrGIS software (version 10.0, ESRI Inc.; Redlands, CA, USA) was used to extract monthly-average NDVI, LSTD, and LSTN, for each pixel of the image. Four indices (minimum, maximum, mean, and standard deviation (SD)) of these monthly-average variable for each year were obtained for each village to capture, albeit crudely, the effects of overall climatic condition and seasonal variation in local climate.

**Distance to the Yangtze River**. Data on the Yangtze River were downloaded from Conservation Science Data Sets of World Wildlife Foundation at http://worldwildlife.org. For each sample village, the Euclidian distance to the Yangtze River was calculated using AcrGIS software.

### Statistical analysis

Ordinary least squares (OLS) regression models were fitted to schistosomiasis data to identify significant environmental covariates. Initially, univariate analyses were conducted and variables with *P*>0.2 were excluded. With the remaining variables, backwards-stepwise regression was conducted using *P* >0.1 as the exit criterion and *P* ≤0.05 as the entry criterion. In the final multivariate model, SD of NDVI, SD of LSTD, and distance to the Yangtze River remained.

A universal spatio-temporal kriging model [22] was fitted to investigate the spatio-temporal pattern of schistosomiasis as well as the effects of environmental factors on the pattern. Let *Y*(*s,t*) denote prevalence of schistosomiasis in village *s* at year *t*. The model assumes that the spatio-temporal process of the prevalence variable is composed of the sum of a trend and a stochastic residual as follows:

Y(s,t)=m(s,t)+ε(s,t) (1)

where *m* is the trend (i.e., a linear function of the covariates), which can be determined by the result of the OLS regression model above, and εis the spatio-temporal correlated stochastic component with zero mean. To ease statistical inference, it is commonly assumed that the zero-mean stochastic part is multivariate normally distributed.

To estimate the spatio-temporal covariance structure of ε, we assume that the variance of ε is constant and that the covariance at sample villages (*s, t*) and (*s* + *h*,*t* + *u*) only depends on the separation distance(*h, u*), where *h* is the Euclidean spatial distance and *u* is the distance in time. Or, simply, we assume ε to be stationary and spatially isotropic. The spatio-temporal covariances are usually described using a spatio-temporal variogram (*γ*), which measures the average dissimilarity between data separated in the spatio-temporal domain using the distance vector (*h, u*) defined as follows:

$$\gamma (h,u)=\frac{1}{2}E{\left[\varepsilon (s,t)-\varepsilon (s+h,t+u)\right]}^{2}$$ (2)

where γ(*h, u*) denotes the semivariance of ε and *E* denotes the mathematical expectation.

In practice, when dealing with real-world data, spatio-temporal variograms are fitted by introducing simplifying statistical assumptions. In this study, we use an inseparable model called “product-Sum” model. This assumes that the spatio-temporal variogram consists of three stationary and independent components:

$$\gamma (h,u)=\gamma_{s}(h)+\gamma_{t}(u)-k\gamma_{s}(h)\gamma_{t}(u)$$ (3)

where γ_*s*_(*h*)and γ_*t*_(*u*)are purely spatial and temporal variograms respectively, and *k* is a real coefficient. The product-Sum model can be seen as a surface with six parameters: two parameters for each variogram (sill and range) and a joint spatio-temporal sill and nugget. In turn, these parameters can be used in spatio-temporal kriging to compute the best linear unbiased predictor (i.e., with minimum expected mean-squared error) for any space-time point where ε (and *Y*) was not observed. The formulas of kriging in the spatio-temporal domain do not differ fundamentally in a mathematical or statistical sense from those of spatial kriging:

$$\overset{⌢}{\varepsilon }(s_{0},t_{0})=c_{0}{}^{T}c^{-1}\bar{\varepsilon }$$ (4)

where *c* is the *n×n* variance-covariance matrix of the residuals at the *n* observation space-time points, as derived from the spatio-temporal variogram, *c*_0_ is a vector of covariances between the residuals at the observation and prediction points, *T* denotes matrix transpose, and $\bar{\varepsilon }$ is a vector of residuals at the *n* observation points. The final prediction of prevalence *Y* at a village (*s_0_, t_0_*) is defined as

$$\hat{Y}(s_{0},t_{0})=\hat{m}(s_{0},t_{0})+\hat{\varepsilon }(s_{0},t_{0})$$ (5)

where $\hat{z}(s_{0},t_{0})$ is the estimated multivariate linear regression trend. For prediction, the endemic area is divided at the 2-km resolution level. A Box-Cox transformation [23] of the crude prevalence is performed to apply the Gaussian model before implementing spatio-temporal kriging. A purely spatial universal kriging was also fitted by year separately for comparative purpose.

The performance of the kriging interpolation would be substantially affected if the spatial stratification is strong and spatial autocorrelation is weak [24]. To ensure this precision, we investigated the spatial heterogeneity of the yearly prevalence of schistosomiasis by employing an indicator of power of determinant (PD) [25]. The PD value, ranging from 0 to 1, quantifies how similar is the spatial distribution of a disease with that of a risk factor. If the PD value is closer to 1, the disease has more similar spatial stratification with that of the factor; if it is closer to 0, the spatial stratifications of the two are quite different. Spatial stratifications of environmental factors were zoned as follows: SD of NDVI and SD of LSTD over the study area were both classified by four equal intervals; distance to the Yangtze River was divided into four buffers: 0~5km, 5~10km, 10~20km, and over 20km.

Cross-validation is applied for assessing the accuracy of the predictions made for prevalence in sample villages as obtained with the spatio-temporal and purely spatial kriging. Specifically, we use leave-one-out cross validation. The method proceeds as follows: one sample village is retained as the validation data for testing the spatio-temporal kriging model, and the remaining sample villages are used as training data to construct the model. This is repeated such that each sample village is used once as the validation data. An indicator of root-mean-square error (RMSE) defined as follows is used to assess the final accuracy of the model:

$$RMSE=\sqrt{\frac{1}{11n}\sum_{t=2000}^{2010}\sum_{i=1}^{n}{\left[\hat{Y}(s_{i},t)-Y(s_{i},t)\right]}^{2}}$$ (6)

where $\hat{Y}(s_{i},t)$ and *Y*(*s_i_, t*) are the predictive and the observed prevalence at the sample village (*s_i_, t*) respectively, and *n* is the number of sample villages.

PD values were calculated using the GeoDetector software freely available at http://www.sssampling.org/Excel-GeoDetector/. Spatio-temporal kriging were implemented in the R package gstat [17], and mapping of predicted prevalence of schistosomiasis and its corresponding variance were performed using the same package as well.

## Results

**Figure 2:**
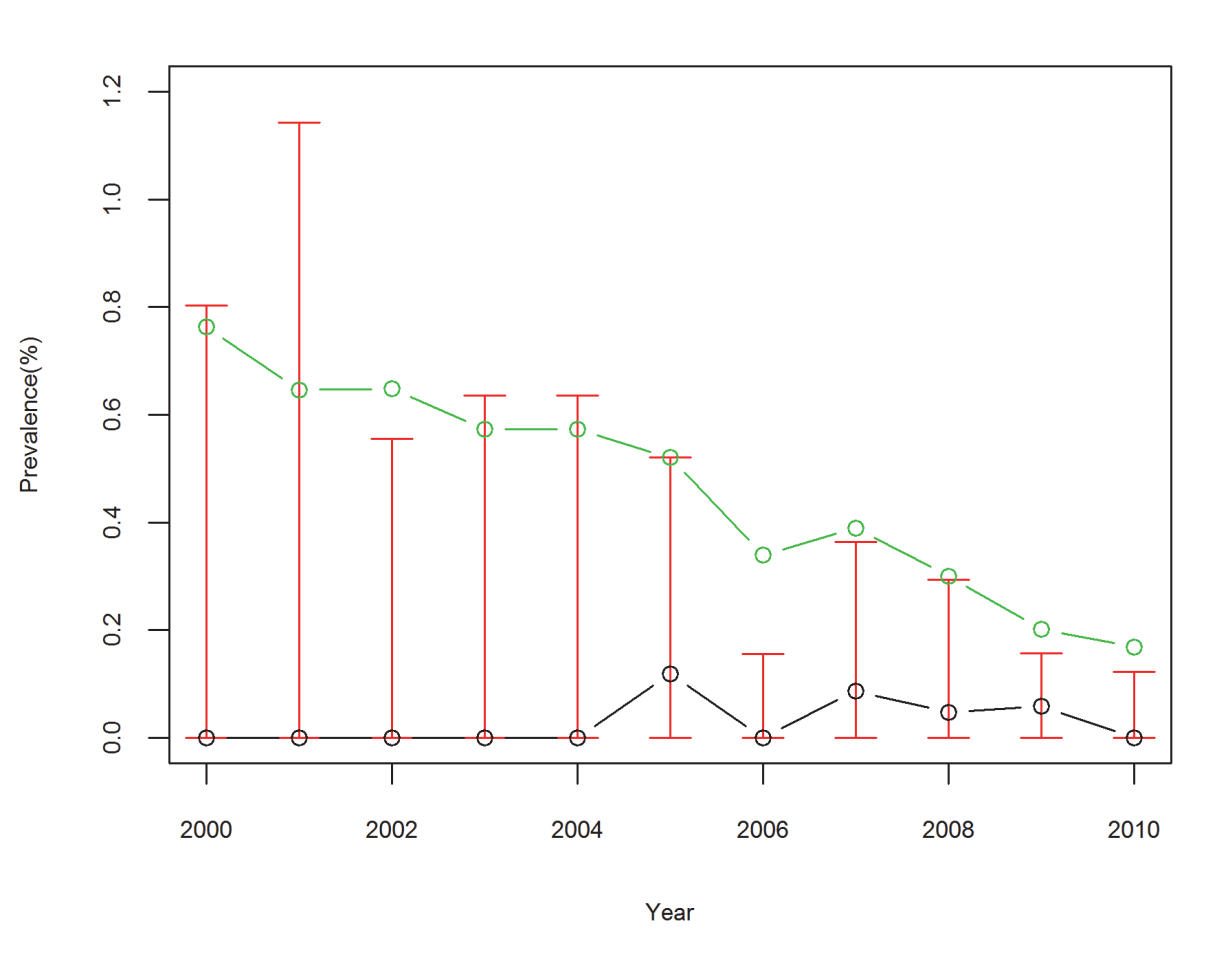
Prevalence of *S*. *japonicum* infection for sample villages in Anhui Province, China, from 2000 to 2010. The red vertical lines denote interquartile range, the green circles denote the mean, and the black circles denote the median.

Fig. 2 gives some statistics about the annual prevalence of schistosomiasis during the study period. The mean observed prevalence generally decreased from 0.76% in 2000 to 0.17% in 2011 and the Kruskal-Wallis test revealed that the mean prevalence significantly differed by year ($\chi^{2}(10,N=1771)=30.47,p<0.01$). This downward trend was accompanied by a decreasing variation in prevalence across villages with the interquartile range (IQR) contracting from 0–0.8/100 in 2000 to 0–0.1/100 in 2010, indicating a decreasing disease burden.

Table 1 shows parameter estimates from the OLS regression model. The results indicate that SD of NDVI, SD of LSTD, and distance to the Yangtze River are significantly associated with *S*. *japonicum* risk. In particular, the infection prevalence increases with decreasing SD of NDVI (coef = -1.21e-04, *p*<0.01), with decreasing SD of LSTD (coef = -5.61e-05, *p*<0.01), and with shorter distance to the Yangtze River (coef = -1.43e-03, *p* = 0.01).

**Table 1:** Ordinary least square (OLS) regression model estimations for schistosomiasis from 2000 to 2010 in Anhui Province, China.

Variable	Estimate	Standard error	t	p	R-squared
SD of NDVI	-1.21e-04	4.50e-05	-2.67	<0.01	0.65
SD of LSTD	-5.61e-05	1.78e-05	-3.15	<0.01	0.65
Distance to the Yangtze River	-1.43e-03	5.80e-04	-2.46	0.01	0.65

SD: standard deviation;

NDVI: normalized different vegetation index;

LSTD: land surface temperature at daytime.

**Figure 3:**
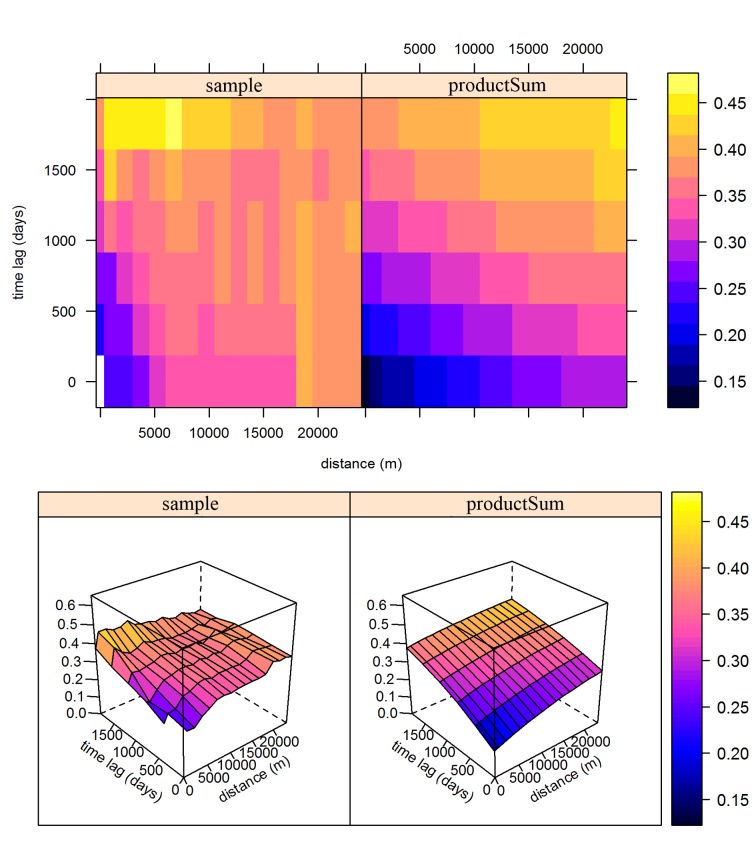
Sample variogram (left) and fitted product-Sum model variogram (right) of residuals from multiple linear regression of schistosomiasis prevalence on SD of NDVI, SD of LSTD, and distance to the Yangtze River. The variogram surface is presented in 2-D (top) and 3-D (bottom).

The left-hand side of Fig. 3 shows the sample residual variogram of the infection prevalence of schistosomiasis, while the right-hand side presents the fitted residual variogram. The rising trend at both spatial and temporal dimension in sample variogram indicates that spatio-temporal correlation is present although the correlation seems not very strong, and therefore, spatio-temporal kriging of residuals is applicable. Table 2 summaries the parameter estimate of the product-Sum variogram model. Note that all variogram components were modeled as exponential function. The range of spatial dependency and temporal dependency is 18 km and 5.48 years (i.e., 2000 days), respectively. The cross-validation results on spatio-temporal kriging and purely spatial kriging yield RMSE of 0.61 and 0.84, respectively, which indicate that the spatio-temporal kriging model has a better predictive ability.

**Table 2:** Parameters of the fitted product-Sum variogram model for the infection prevalence of schistosomiasis from 2000 to 2010 in Anhui Province, China.

	Nugget	Sill	Range
Spatial	---	0.21	18 km
Temporal	---	0.39	2000 days
Space-time	0.14	0.39	---

Table 3 presents PD values of the environmental factors for each year, which help to investigate spatial heterogeneity of the prevalence of schistosomiasis. The PD values of SD of NDVI range from 0.15 to 0.20 with the mean of 0.17, those of SD of LSTD vary from 0.13 to 0.18 with the mean of 0.15, and those of distance to Yangtze River change from 0.26 to 0.33 with the mean of 0.28.

**Table 3:** Power of determinant (PD) values of the environmental factors for each year.

Factors	2000	2001	2002	2003	2004	2005	2006	2007	2008	2009	2010
SD of NDVI	0.15	0.18	0.17	0.16	0.15	0.20	0.18	0.18	0.17	0.16	0.17
SD of LSTD	0.13	0.14	0.18	0.15	0.15	0.18	0.14	0.15	0.14	0.15	0.16
Distance to the Yangtze River	0.28	0.30	0.29	0.27	0.26	0.33	0.26	0.27	0.30	0.29	0.28

SD: standard deviation;

NDVI: normalized different vegetation index;

LSTD: land surface temperature at daytime.

**Figure 4:**
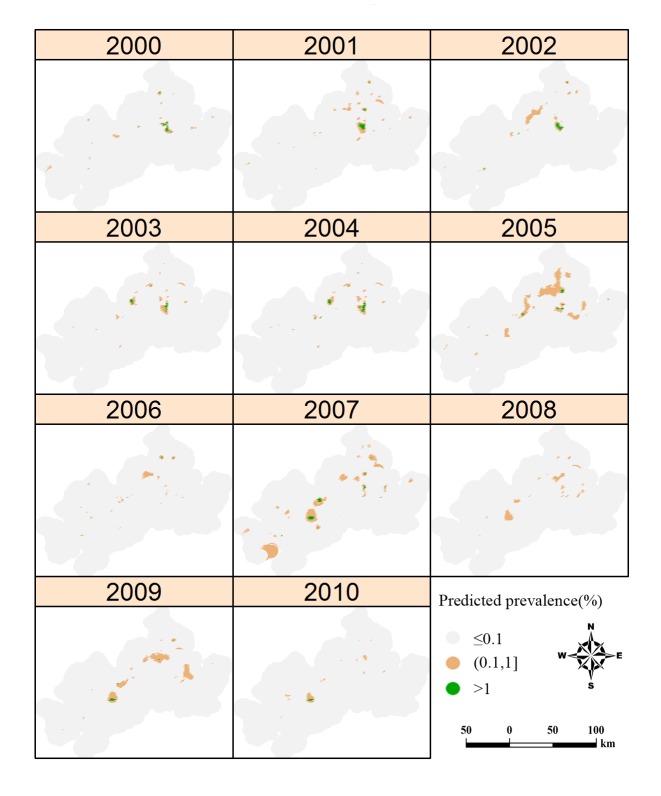
Annual predicted prevalence of schistosomiasis in Anhui Province, China, from 2000 to 2010. Predictions were conducted only in endemic areas. The maps were created using the R package gstat.

**Figure 5:**
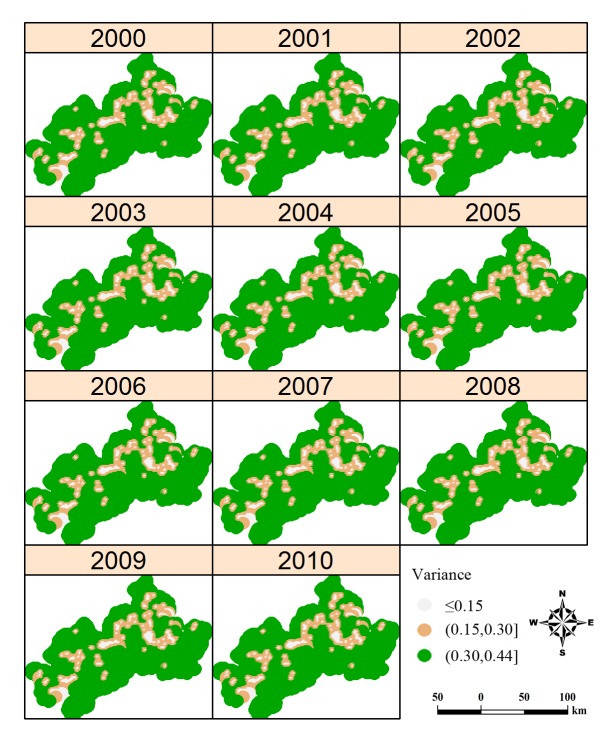
Annual uncertainties of the predictions of schistosomiasis in Anhui Province, China, from 2000 to 2010. Predictions were conducted only in endemic areas. The maps were created using the R package gstat.

Fig. 4 displays the annual map of predicted prevalence for *S*. *japonicum* infection and it can be seen that the infection prevalence is generally low, namely, most areas with prevalence below 0.1% and very limited areas with prevalence over 1%. The infection risk showed a focal spatial pattern and the pattern fluctuated temporally with a peak (the largest spatial extent with prevalence over 0.1%) in 2005 and then contracted gradually but with scattered distribution until 2010. Note that clusters of schistosomiasis risk mostly occurred along the Yangtze River. Fig. 5 represents corresponding estimates of the variance of the predictions. The maps present similar patterns across the study period: a lower level of uncertainty is apparent in locations close to sampled villages while a higher level of uncertainty is present in locations distant from sampled villages. The prediction uncertainty is generally low over the study area.

## Discussion

This study demonstrates the use of a spatio-temporal kriging model in assessing how environmental factors affect the outcome of human schistosomiasis based on spatio-temporally correlated disease data. Our results confirmed spatio-temporal differences in the infection risk and the important role of environmental factors in explaining the variations. The predicted risk maps, in return, provide an empirical basis for identifying priority areas when implementing schistosomiasis controls locally.

Schistosomiasis is a water-borne disease and its transmission is strongly associated with environmental factors. We, therefore, considered three key elements that characterize schistosomiasis transmission [26,27,28], namely, temperature, wetness, and access to infected water, using LSTD, LSTN, NDVI, and distance to the Yangtze River. Many studies [29,30,31,32,33] had explored effects of these elements, but seldom did those use the four indices (minimum, maximum, mean, and SD) of climatic factors to account for effects of overall climatic condition and seasonal variation in local climate. Our risk analysis showed that seasonal variation of LSTD and NDVI, and the Yangtze River were significantly, negatively correlated with the risk of *S*. *japonicum*. There is a plausible, biological explanation for these associations as discussed below.

The explanation for the negative association between seasonal variation of LSTD and schistosomiasis risk derives from the parasite’s life cycle, of which several stages (i.e., the excreted egg, miracidium, sporocyst, and cercaria) require fresh water environment. Previous studies have shown that the development of the parasite residing in the intermediate host snail is closely related to the environmental temperature, e.g., large seasonal temperature differences (characterized by higher SD) would hamper the development of miracidia into cercariae and the situation worsens if the thermal limits are exceeded [34,35]. The parasite cannot complete its life cycle optimally in areas with larger temperature differences, and hence less cercariae are released into freshwater environments. As the cercaria is the infective stage for both humans and mammalian reservoir hosts, the disease transmission intensity decreases. NDVI maps, indicating the amount of vegetation present at each location, have been widely and successfully used for prediction of intermediate host snails of schistosomiasis [31] and are often used as a proxy for suitable *O*. *hupensis* habitats. An area with higher SD of NDVI indicates the vegetation coverage is not constant and hence it is not ideal for formation of snail habitat. The distance to the Yangtze River can be seen as a proxy of exposure (due to increased water contact). Individuals living near the shore are more likely to risk contact with water containing infected snails as a result of their professional work (e.g. agricultural activities, fishing, etc.) and life style (e.g., cleaning and swimming). In addition, frequent seasonal flooding events might also increase their exposure to cercariae.

Parameters of fitted residual variogram of the infection prevalence of schistosomiasis can characterize the spatial and temporal variation of the disease. The spatial range of 18km suggested that the spatial correlation become negligible after 18km, and such distance implied that transmission occurred between villages rather than within and around them. On the other hand, the temporal range of about 6 years indicated that temporal correlation become negligible after this range, and such a long period reflected that the burden of schistosomiasis should not vary greatly for each year and was probably related to the low infection rate as a result of implementation of schistosomiasis control strategies. The rising trend of the sample residual variogram (as shown by the 3-D plot in Fig. 3) indicated that spatio-temporal correlation was present in schistosomiasis, though it is not very strong, after adjusting the current environmental factors. This mild spatio-temporal correlation suggested that the spatio-temporal pattern in schistosomiasis risk is probably already captured by the environmental factors.

Kriging interpolation is principally based on spatial/spatio-temporal autocorrelation, the precision of which would be poor if the spatial stratification is strong and spatial autocorrelation is weak. The mean PD value of 0.17 for distance to snail habitat indicated that SD of NDVI explained 17% of the variation of schistosomiasis; similarly, SD of LSTD and distance to snail habitat explained 15% and 28% of the variation of the disease, respectively. The findings implied that prevalence of schistosomiasis showed week spatial heterogeneity within the buffers. Combined with ST correlation, the week spatial heterogeneity of the disease over the study area during the study period justified the employment of spatio-temporal kriging.

The spatio-temporal variations of schistosomiasis risk shown in Fig. 4 can be explained by the schistosomiasis control strategies implemented in the study period. In the early 1990s, the Chinese government launched a 10-year World Bank Loan Project (WBLP) on schistosomiasis control [36], strongly based on large-scale chemotherapy but with additional intervention activities such as health education, chemical control of snails, and other environmental exposure modifications. The disease, however, rebounded shortly after the conclusion of WBLP in 2001 [37,38]. This rebound can be seen from maps in 2002–2005. In order to deal with the rebounding trend, a revised strategy, aimed at reducing the role of bovines and humans as infection sources and based on integrated measures, was implemented from 2005 [39]. In addition to chemotherapy and health education, water buffaloes and cows were replaced by tractors and the integrated program also included such strategies as treatment of night-soil and provision of piped, safe water [40], keeping domestic animals in barns [41], and reduction of snail habitats through the construction of water conservancy projects [41]. However, the scattered distribution of schistosomiasis risk shown in maps of 2007 to 2009 suggested that the integrated strategy could not effectively compress the spatial extent of the disease, indicating there are still large populations at risk.

As a reflection of rebound trend of schistosomiasis, infected *O*. *hupensis* snails are still found in certain locations along the Yangtze River [42], and there is concern that they might spread further, possibly resulting in extensive re-emergence of infections among people and domestic animals. Since control measures are limited to bovines and humans and more than 40 species of mammalians can serve as potential zoonotic reservoirs, the infectious-source measures can’t block the life cycle of the parasite completely [43]. Furthermore, the integrated strategy is expensive as it involves many diverse activities while budgets on schistosomiasis control may probably be reduced in the foreseeable future. A less costly, but still effective and sustainable control strategy is urgently needed. Targeting the snail habitats within areas of high schistosomiasis risk can be a way out as the amphibious *O*. *hupensis* is the only intermediate snail host and may, therefore, be the weak link in the parasite’s life cycle. Our analysis provides an empirical basis for identifying priority areas. As seen in Fig. 4, areas with relatively high risk (i.e., prevalence > 0.1%), especially those areas with constant clusters of risk, would definitely be a priority for targeting schistosomiasis control in local regions.

Some limitations in our study deserve further discussions. Firstly, the slightly rising trend of the sample residual variogram in Fig. 3 is probably due to lack of other risk factors, which indicates that more risk factors should be considered. Therefore, in addition to the current environmental factors (i.e., LST, NDVI, and the Yangtze River), many other factors, such as landscape metrics, socio-economic impacts, interventions, etc., should be warranted in further studies. Second, spatio-temporal kriging model relies on an assumption of stationarity. This assumption is appropriate when mapping disease over small areas, but might be questionable over wide areas, such as a country [44]. To investigate the effects of non-stationarity is interesting and challenging topic and should be considered in further studies. Finally, the specificity of serological assays and the sensitivity of stool examination tests are not perfect [45]. The generally low levels of infection with *S*. *japonicum* in recent years result in uncertainty both with regard to sensitivity and specificity of infection [46]. Modeling with diagnostic errors should be considered in future studies.

In summary, this study investigated a region where schistosomiasis endemic remains of public health and economic significance and might spread as infected snails are still found. Combined with techniques of GIS and RS, the spatio-temporal kriging model disclosed the spatio-temporal patterns of *S*. *japonicum* infection, which, we believe, helps to facilitate and enrich risk modeling of the disease. The results can be used to identify priority areas where control efforts should be taken. Compared to the ongoing costly integrated strategy with infectious-source controlling as an emphasis, targeting snails (the only intermediate host in the parasite’s life cycle) and taking corresponding effective actions (e.g., mollusciciding and environmental modification) in these priority areas would be sustainable in schistosomiasis control in the long term.
